# Variability of Non-Polar Secondary Metabolites in the Red Alga *Portieria*

**DOI:** 10.3390/md9112438

**Published:** 2011-11-21

**Authors:** Dioli Ann Payo, Joannamel Colo, Hilconida Calumpong, Olivier de Clerck

**Affiliations:** 1 Phycology Research Group, Ghent University, Krijgslaan 281, S8, 9000 Ghent, Belgium; E-Mail: jm2marine@yahoo.com; 2 Institute of Environmental and Marine Sciences, Silliman University, Dumaguete City 6200, Philippines; E-Mail: hpcalumpong@yahoo.com

**Keywords:** *Portieria*, secondary metabolite, variation, cryptic species, life-history stages

## Abstract

Possible sources of variation in non-polar secondary metabolites of *Portieria hornemannii*, sampled from two distinct regions in the Philippines (Batanes and Visayas), resulting from different life-history stages, presence of cryptic species, and/or spatiotemporal factors, were investigated. PCA analyses demonstrated secondary metabolite variation between, as well as within, five cryptic Batanes species. Intraspecific variation was even more pronounced in the three cryptic Visayas species, which included samples from six sites. Neither species groupings, nor spatial or temporal based patterns, were observed in the PCA analysis, however, intraspecific variation in secondary metabolites was detected between life-history stages. Male gametophytes (102 metabolites detected) were strongly discriminated from the two other stages, whilst female gametophyte (202 metabolites detected) and tetrasporophyte (106 metabolites detected) samples were partially discriminated. These results suggest that life-history driven variations, and possibly other microscale factors, may influence the variation within *Portieria* species.

## 1. Introduction

Natural products, and in particular secondary metabolites, have been the focus of study in many marine macroalgae. Seaweeds interact with their environment utilizing a rich variety of secondary metabolites [1–4]. These chemical compounds have no explicit role in the internal metabolism of the organisms [1,5,6] but serve as defense mechanisms against grazers, competitors, fouling organisms and pathogens [7–9]. The compounds are localized in specialized cells, have relatively low molecular weights (<3000 Daltons), are structurally diverse, often halogenated, and exist in low abundance (often <1% of total carbon) [1,2,6,10–12].

Secondary metabolites have drawn wide attention because of their pharmaceutical potentials, chemotaxonomic and ecological importance [13–16]. Macroalgae produce a wide range of compounds such as terpenes, phenols, fatty acids, lipopeptides, amides, alkaloids, terpenoids, lactones, pyrroles and steroids [9,15,17]. Red seaweeds belonging to the family Rhizophyllidaceae have been found to be especially rich in secondary metabolites, harboring a variety of halogenated monoterpenes. The tropical, Indo-Pacific genus *Portieria* (synonyms *Chondrococcus* and *Desmia*), is a prolific source of these halogenated compounds [11,12,18–24], about 80 of which have been isolated (Table S1). Among the most interesting of these compounds is halomon, a monoterpene with anti-tumor properties, isolated from samples collected in Batanes, Philippines [22,23]. However, the inconsistent availability of this compound from natural populations prevented further drug development.

Previous studies have mainly focused on discovery and pharmaceutical potentials of chemical compounds in *P. hornemannii*. Only a few studies have addressed inter- or intraspecific variation in secondary metabolite composition. Puglisi and Paul [25] tested the carbon/nutrient hypothesis, which postulates that the secondary metabolites produced by a certain alga are dependent on the nutrient availability. They found that variation of ochtodene concentrations in *P. hornemannii* cannot be attributed to nitrogen and phosphorus availability but suggested instead that light was a contributing factor. Matlock *et al.* [24] demonstrated strong site-to-site differences, variation within populations, and limited evidence for temporal variation in apakaochtodene levels. Some authors have emphasized the necessity to genotype the organisms in order to better understand the mechanisms that regulate the production of specific natural products [5,26–28].We found evidence for a large number of cryptic species within *Portieria* in the Philippines [29], but the effect of intraspecific genetic variation in relation to secondary metabolites has not been tested to date.

This study aims to understand if non-polar secondary metabolites of *Portieria* plants vary quantitatively or qualitatively between cryptic species and among life stage within species. We employed metabolite fingerprinting and multivariate analyses of chromatograms of extracts of samples collected in the Philippines. The large number of compounds produced by *Portieria*, the lack of commercial standards and the scarcity of compound identification resources (mass spectral databases) dedicated to marine secondary metabolites makes metabolite fingerprinting the most feasible analytical method to define variation between cryptic species and among life-history stages within species. In contrast to target analysis, metabolite fingerprinting does not separate individual metabolites, but instead compares spectra of whole extracts using multivariate statistics [30]. Metabolite fingerprinting is particularly useful for rapid classification of samples [31]. Our specific aims were to evaluate: (1) if there is variation of non-polar secondary metabolites between the gametophyte and tetrasporophyte life-history stages of *Portieria*, (2) if metabolite variation is due to the presence of phylogenetically distinct cryptic species, and (3) if patterns of variation are observed on a geographical and temporal level.

## 2. Results and Discussion

### 2.1. Cryptic Diversity

**Figure 1:**
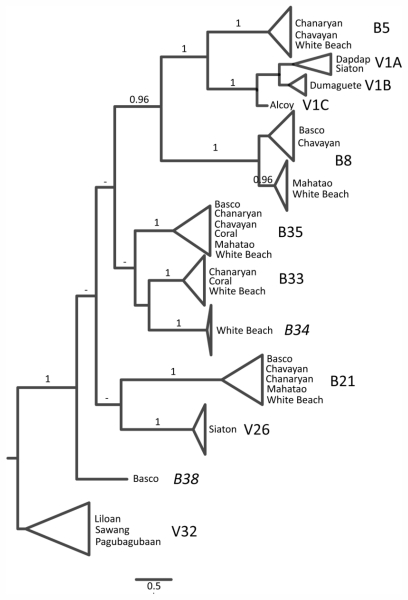
Phylogenetic tree reconstructed using Bayesian inference based on the mitochondrial *cox*2–3 spacer of *Portieria* specimens collected from Batanes (B) and Visayas (V) Islands in the Philippines. Branch support (posterior probabilities) ≥ 0.5 are indicated at the branches. The eleven clades represent cryptic lineages, likely equivalent to species. Species B34 and B38 were not included in the chemical analysis.

Phylogenetic analyses of 152 *cox*2–3 sequences revealed 12 clades of closely related sequences, preceded by long well-supported branches. Seven lineages include specimens from Batanes and five clades are restricted to the Visayas (Figure 1). These clades will further be regarded as species. Details on cryptic diversity and species delimitation are reported in Payo [29].

### 2.2. Identification of Compounds in Non-Polar Extracts

Owing to the lack of standard compounds and public repositories of mass spectral data specific to marine metabolites, the identity of the compounds was investigated only by comparing the mass spectra and the Kovats retention indices to those deposited in publicly available databases (MassBase, Pherobase, Lucero Library and the free limited NIST database in the MS Search Program and AMDIS). Identifications were based on a minimum of 80% similarity of mass fragments. Using a retention index calculator [32], plant volatiles with the nearest Kovats Index (KI) to that of the compounds in the samples were retrieved. The KI used for compound identification is based on a DB5 capillary column similar to the non-polar, low bleed HP-5MS column used for GC/MS in this study. The Lucero KI based identification was verified with the mass spectra and KIs listed in Pherobase. Comparison with mass spectra of compounds found in *Portieria* in the literature [11,12,33] did not reveal any positive identification. Examination of mass fragments did not show any similarity of components with that of halomon’s mass spectral fragments as reported by Egorin *et al.* [33].

### 2.3. Metabolite Fingerprinting

#### 2.3.1. Variation Between Life Stages

**Figure 2:**
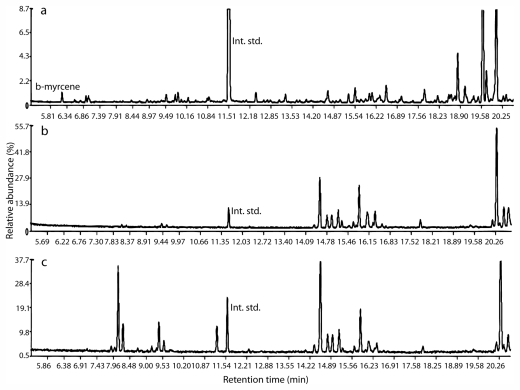
Portion of GC-MS total ion chromatograms of non-polar extracts of *Portieria* samples: (**a**) male gametophyte from Bantayan (Dumaguete), V1; (**b**) White Beach, Batanes, B21; (**c**) Liloan, V32. β-myrcene, a precursor of many halogenated monoterpenes, was detected only in the Bantayan specimens. Naphthalene is used as an internal standard.

Plants used for this study belonged to a single species V1B (Figure 1) and were collected on the same day from a single site in Bantayan, Dumaguete. A total of 202 non-polar compounds were detected from gametophytes and tetrasporophyte samples by GC-MS (Table S2). Using an external standard and the databases, the first peak of each sample (RT = 6.18 min) was confirmed as β–myrcene (Figure 2a). Of the remaining compounds, only 11% (22) were identified using NIST and MassBase. Based on the NIST and MassBase identifications, five compounds were monoterpenes. Examination of the patterns of parent ions showed ten halogenated compounds containing one Cl or one Br. Two hundred compounds had a KI ranging 993–1969. Some peaks with succeeding retention times shared the same KI but had a different mass spectra composition. NIST and MassBase did not share the same identification of peaks except for β-myrcene.

KI for the last two peaks were not computed since it occurred later than the last alkane standard (C20 at 31.226 min) that was used for KI calculation. We also explored the potential of the Lucero library and Pherobase to identify compounds that *Portieria* might have in common with land plants. The Lucero library retrieved the nearest KIs of plant volatiles. Some of these identifications were verified at Pherobase, but mass spectra composition of the unidentified peaks was not similar to the spectra of the compound name retrieved from the database. Furthermore, we also tried to retrieve compounds with a certain KI and examined the corresponding spectra. β-Myrcene has a KI of 993.543 in this study. Pherobase listed 991 and 994 as KIs for β-myrcene in a DB5 column. Nine compounds listed in the database have a KI of 993.

**Figure 3:**
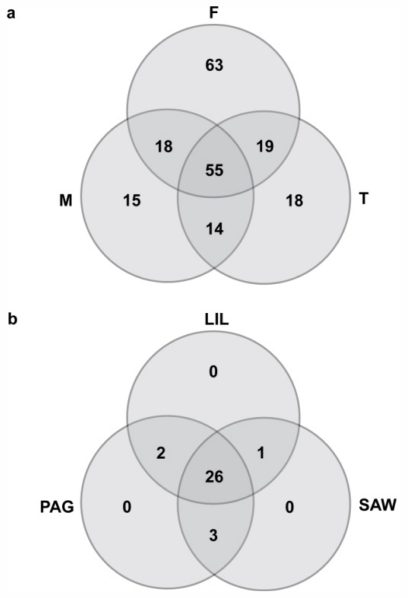
Frequency distribution indicating number of shared and unique non-polar secondary metabolites: (**a**) within life-history stage of species V1B from Bantayan, female (F), male (M) and tetrasporophyte (T); (**b**) among sites of species V32 collected in Sawang (SAW), Liloan (LIL) and Pagubagubaan (PAG).

The Lucero library lists KI 993 for β-myrcene. With the large number of metabolites retrieved, this method of verification proved cumbersome especially since Pherobase currently does not allow automated mass spectral and KI comparison. Of the 202 compounds detected, 155 compounds were present in the female samples, 102 in male plants and 106 in tetrasporophytes (Figure 3a). There were 55 compounds that were shared by all life stages. Sixty-three compounds were found exclusively in female gametophyte extracts, 15 in male gametophyte extracts, and 18 in tetrasporophyte extracts. There were 14 compounds common to male and tetrasporophyte extracts. The male and female gametophyte shared 18 compounds while female and tetrasporophyte shared 19 compounds. A precursor compound of the monoterpenes, myrcene, was detected in all of the life stages but not in all replicates.

**Figure 4:**
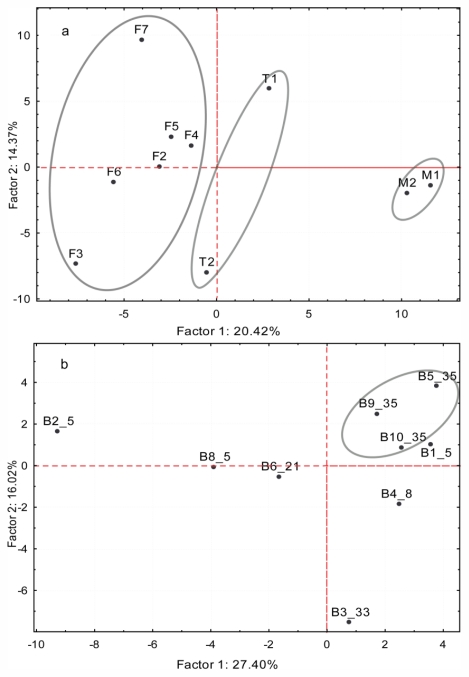
Principal component analysis of GC-MS standardized relative abundance datasets which includes the compounds detected in a 67-min run of *Portieria* extracts. (**a**) Male gametophyte (M1-2) samples are clearly discriminated occurring at the positive end of the plane while female gametophyte (F2-7) and tetrasporophyte samples (T1-2) are only partially discriminated by Factor 1; (**b**) Batanes dataset includes five cryptic species. The clustering of B35 replicates and the scattered pattern of B5 suggest variation in component compounds exists between species but at the same time suggested that variation within species can occur.

We applied a principal component analysis (PCA) on the standardized relative abundances to determine if metabolites differ among life stages (Figure 4a). The matrix contained 202 compounds (active variables) and ten samples (active cases: six female gametophytes, two male gametophytes, and two tetrasporophytes). All samples were collected on the same day from a single site, and belong to a single species. Figure 4a shows the projection of the cases on the factor plane. Factor 1 and factor 2 accounts for 20.42% and 14.37% of the variation, respectively. Factor 1 clearly discriminated the male gametophytes from the others, whilst the female gametophytes and tetrasporophytes, although separated, were more closely aligned. Factor 2 does not discriminate between life stages but points towards additional variation within life stages.

#### 2.3.2. Variation Between Cryptic Species

A total of 67 compounds were detected from nine samples (three technical replicates) by GC-MS (Table S3). These samples belonged to the five species B8, B5, B21, B33 and B35, as determined by DNA sequence analysis. A sample consists of pooled extract of eight individuals belonging to one species as verified by DNA analysis. β-Myrcene was not detected in any of the samples (Figure 2b). Unfortunately the three databases used (NIST, MassBase, Lucero library) did not give identical identifications of compounds. NIST identified four compounds appearing at different times as 2,2-dimethyl-3-hexanone. Comparison of mass spectra with MassBase resulted in the retrieval of straight and branched alkanes and alkenes. Compounds with retention times from 8.11 to 30.69 min had retention indices from 1065 to 1965. No KIs were computed for the rest of the compounds. Comparison of computed KIs with those in the Lucero library indicated compounds from these samples contained monoterpenes, sesquiterpenes, esters, fatty acids and alcohols. NIST, MassBase and the Lucero databases did not share the same identification of compounds. Visual examination of parent ions showed only two compounds to be halogenated containing either one Cl or one Br.

The number of compounds detected varied between the five species (Table 1) and among biological replicates. Species B35 had the highest number of compounds detected (60/67), species B33 contained 51/67 compounds detected, species B8 and B5 both had 57/67 compounds detected and species B21 had the least number of compounds (49/67). There were 21 compounds that were detected in all nine samples, while 33 compounds were present in all five species.

To detect distinct groupings, we conducted a PCA based on the standardized relative abundances of the nine samples using the 67 compounds as variables (Figure 4b). The first principal axis accounted for 27.4% of the variance while the second 16.0%. Plotted scores from the PCA did not show distinct interspecific clusters. The largest variation was displayed by specimens belonging to species B5.

#### 2.3.3. Spatial and Temporal Variation

Six sites in the Visayas (Sawang, Pagubagubaan, Dapdap, Siaton, Liloan and Bantayan (Dumaguete); *n* = 44) were sampled from 2007 to 2009 and investigated using Spectconnect to determine if seasonality or geography explained the variation in secondary metabolites. A total of 33 compounds were detected from these samples (Table S4), however, β-myrcene was not detected (Figure 2c). As in previous datasets, the three databases (NIST, MassBase, Lucero library) used did not give identical identifications of compounds. Comparison of mass spectra with MassBase resulted in the retrieval of eleven compounds, most likely straight and branched alkanes and alkenes. Many of the peaks detected eluted later than the standard, icosane (C20), hence were not included. Comparison of computed RIs with those in Lucero library retrieved the following compound classes: monoterpenes, sesquiterpenes, esters and fatty acids. NIST library identified two compounds, 3-hexanone, 2,2-dimethyl- and disilane. No halogenated compounds were detected.

**Figure 5:**
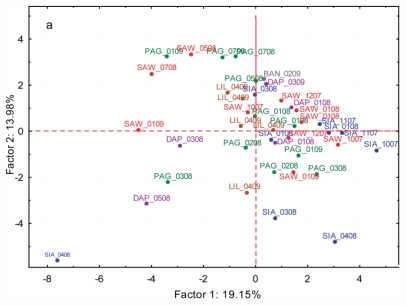

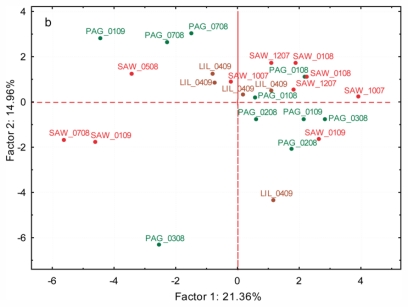
Principal component analysis of GC-MS standardized relative abundance datasets for detecting possible spatial and temporal patterns in: (**a**) the Visayas species; (**b**) the Visayas samples belonging to species V32. Letters indicate sampling site (DAP: Dapdap; PAG: Pagubagubaan; SAW: Sawang; SIA: Siaton) and numbers indicate month and year of sampling.

PCA was conducted using the standardized relative abundance file generated by Spectconnect (*n* = 44) using the 33 compounds as variables (Figure 5a). The first and second PCs accounted for 17.87% and 17.78% of the variance, respectively. No clear groupings could be deduced based on species, sampling period, or sampling site. Most of the samples clumped except for one Siaton and two Bantayan samples, which were clearly separated from the rest. A PCA excluding these three samples slightly spread the rest of the samples but likewise did not show any recognizable clusters (Figure 5a). A separate PCA analysis was conducted on samples belonging to the same species, V32, from Sawang, Pagubagubaan, and Liloan (Figure 5b). The first and second PCs accounted for 21.36% and 14.96% of the variance, respectively. Again, no clusters based on site or period of collection were detected.

### 2.4. Patterns of Secondary Metabolite Distribution

Distribution of secondary metabolites in marine macroalgae exhibit macroscale and microscale patterns [34]. Macroscale patterns include global patterns within or across algal taxa, patterns within a specific habitat, and patterns correlated with biotic and abiotic factors. Microscale patterns are related to molecular and biochemical processes within an algal thallus, the spatial distribution of compounds within an algal thallus and temporal responses (short- and long-term responses). In *Portieria* neither macro- nor microscale patterns of secondary metabolite variation have been fully explored.

Although three decades of studies have been dedicated to the isolation of new compounds only two have investigated the variation of these, specifically ochtodene and apakaochtodene A and B [24,25]. However, a possible drawback related to these studies is the fact that individual compounds are not mutually independent but are linked via biological pathways as is the case for β-myrcene, which is the likely precursor of many monoterpenes [35], and whose presence influences the presence or absence of other halogenated monoterpene components. Hence in order to understand general or specific metabolite patterns, knowledge of the presence and absence of progenitor compounds is essential. The present study has successfully described the variation of the non-polar secondary metabolites of *Portieria* on a macroscale level using metabolite fingerprinting.

Reports on intraspecific variation of secondary metabolites in macroalgae have been based on variations within individuals, between individuals of a population and among geographically isolated populations [36,37]. Our results suggest substantial intraspecific variation among the life-history stages of *Portieria*. Male gametophytes form a clearly distinct group, while a partial discrimination was detected between female gametophytes and tetrasporophytes (Figure 4a). Furthermore, our results show that female gametophytes are chemically richer in terms of the number of secondary metabolites (Figure 3a), possibly providing protection to the carposporophyte from grazers. Many of the compounds detected appear to occur exclusively to a certain life-history stage while a relatively small group of compounds is shared among life-history stages. Similarly, male gametophytes of *Asparagopsis armata* were found to have lower concentration of the secondary metabolite bromoform compared to female gametophytes [37]. It has also been observed that the carposporophyte phase of *A. armata* was the least consumed life-history phase by the seahare, *Aplysia parvula* [37]. Likewise, in the brown alga *Dictyota menstrualis*, female gametophytes were found to produce more diterpenes compared to the other life-history stages [38].

Comparative analyses of the five cryptic species from Batanes (B5, B21, B33, B35, and B8) showed variation between, as well as within species (Figure 4b). Intraspecific variation is even more pronounced in the Visayas dataset (Figure 5). Neither species specific nor spatio-temporal patterns were evident.

In addition, a separate PCA including only specimens of species V32 (Figure 3b) did not elucidate patterns based on collection site or period. The results suggest that life history related variations combined with microscale factors have a large influence on the variation within *Portieria* species. Pelletreau and Targett [34] pointed out that the existence of inducible and activated defenses complicates the search for universal patterns of secondary metabolites and continues to highlight the importance of localized phenomena. We conducted a preliminary grazing experiment in outdoor flow-through aquaria to assess the influence of the herbivore (*Aplysia*) on the production of metabolites in *Portieria*. However, these efforts were beset by two factors: death of plants due to high water temperature and continuous development of microscopic *Aplysia* larvae on field collected plants undergoing acclimatization. Future grazing experiments will attempt to eliminate these factors. In addition more extensive sampling should confirm the patterns observed during the present study.

Finally, there was difficulty in achieving unequivocal identification of compounds in this study unveiling the need for mass spectral and KI databases dedicated solely to marine secondary metabolites. Such repositories will assist in the identification of previously reported compounds for investigations mainly focused on rapid detection and understanding of chemical patterns in marine organisms. In doing so, future studies will not just be focused in isolating and naming novel compounds as had been the trend in *Portieria* but will also seek to answer questions of biological or ecological relevance. Furthermore, the presence of cryptic species in *Portieria* highlights the need to genotype organisms ensuring that correct species identification is correlated with metabolite fingerprint, enabling more targeted approaches in future studies.

## 3. Experimental Section

### 3.1. Collection and Storage

Field samplings were performed at different periods from several sites in the Philippines (Figure S1). Plants were collected by snorkeling and SCUBA, diving depths ranging from 1–20 m. For secondary metabolite characterization of the different life-history stages of *Portieria*, plants were collected randomly from Bantayan near Dumaguete (Figure S1c) on December 29, 2009. To allow both chemical characterization and life-history stage verification plants were blotted dry and stored over silica.

Plants for secondary metabolites analysis between species were collected from four sites in Batanes (Basco, White Beach, Coral, and in Chavayan) (Figure S1a,b) on April 23–26, 2009. Plants were preserved in 95% ethanol in the field and stored at −23 °C afterwards. Sampling for seasonal and spatial comparison of secondary metabolites were conducted from six sites in the Central Visayas (Dapdap, Siquijor, Siquijor; Pagubagubaan, San Juan, Siquijor; Sawang, San Juan Siquijor; Bantayan, Dumaguete, Negros Oriental; Siaton, Negros Oriental; Liloan, Santander, Cebu (Figure S1a,c) from 2007 to 2009. For the first four sites, plants were collected on a monthly basis when present and when abundant enough to afford a good amount for extraction. For sites close to the Silliman Marine Laboratory, plants were immediately packed and stored at −23 °C upon arrival. When immediate freezing was not possible, plants were preserved in 95% ethanol in the field and stored at −23 °C. Frozen plants were rinsed in freshwater or in ethanol and blotted dry.

As *Portieria* cannot be distinguished morphologically, species identification was based on DNA sequence data (see below). For DNA characterization, thallus clippings were collected from specimens and stored in silica while in the field.

### 3.2. Extraction

#### 3.2.1. Life-History Stages Comparison

*Portieria* has a tri-phasic isomorphic life history which includes free-living haploid male and female gametophytes, a diploid carposporophyte attached to and dependent on the female gametophyte, and a free-living diploid tetrasporophyte. Silica-dried specimens were examined under the microscope and segregated into different life-history stages (male gametophytes, female gametophytes, and tetrasporophytes). Biological replicates, each about 1 gram dry weight, were prepared for every life-history stage. Tissues were ground using mortar and pestle, dissolved in 6 mL dichloromethane (DCM) containing 50 μg·mL^−1^ naphthalene, and vortexed for a few seconds. The DCM extracts were allowed to stand at room temperature for 24 h in screw-cap tubes. The extracts were decanted and transferred to air tight screw-cap tubes and stored at −23 °C. The tissues were steeped in DCM for another 24 h. First and second extracts of a sample were pooled and filtered using Whatman GF/C. The combined extracts were loaded onto pre-conditioned SPE tubes containing 10 g of silica (Silicycle SiliaFlash^®^ G60, 60–200 μm, 60 Å). Non-polar compounds were eluted using 12 mL hexane. The eluate was concentrated to 0.5 mL under N_2_ gas. Three 100 μL replicates were taken from this stock and used as technical replicates in the GC-MS analysis.

#### 3.2.2. Species Comparison

The samples were extracted individually. Tissue was ground using a mortar and pestle and extracted twice in 6 mL DCM overnight. Extracts were pooled, loaded onto 2 g silica and eluted using 6 mL of hexane. Eluates of eight samples belonging to the same species, approximately weighing 10–26 g, were pooled and evaporated down to 100 μL. To assess consistency in the GC-MS analyses, three technical replicates were prepared for each sample.

HPLC solvents for extraction and chromatography (DCM and *n*-hexane) were sourced from Mallinckrodt or HiperSolv Chromanorm). Naphthalene (Fisher) and β-myrcene (Sigma) were used as internal and external standards, respectively.

#### 3.2.3. Seasonal and Spatial Comparison

For seasonal analysis, plant material was ground using mortar and pestle. Twenty grams of ground material was extracted in 20 mL DCM for 24 h at room temperature. The material was extracted a second time in 20 mL DCM and the extracts were pooled. To measure the amount of green oil that was generated, one of the two pooled extracts was evaporated to dryness using nitrogen. The oil was weighed and redissolved in 20 mL DCM. The other extract was evaporated down to 20 mL. Both extracts were loaded in SPE columns containing 10 g of silica (Silicycle SiliaFlash^®^ G60, 60–200 μm, 60 Å). Non-polar compounds were eluted from the column with 20 mL hexane and again reduced to 100 μL. A separate aliquot of ground plant tissue was used to determine dry mass.

### 3.3. Phylogenetic Analysis

Assignment of the sampled specimens to cryptic species was based on phylogenetic analysis of the mitochondrial *cox*2–3 spacer region. The phylogeny includes specimens collected from five sites (Basco, Chanaryan, Coral, Mahatao, Chavayan) in Batanes and six sites (Dapdap, Siquijor; Sawang, San Juan, Siquijor; Pagubagubaan, San Juan, Siquijor; Malo, Siaton; Bantayan, Dumaguete; Liloan, Santander) in the Visayas, Philippines. Species delineation follows the rational outlined in Payo *et al.* [29]. DNA extraction, PCR amplification, sequencing and sequence alignment were performed as described in Payo *et al.* [29].The alignment of 152 *Portieria* sequences was 345 bp long. Bayesian inference (BI) of phylogeny was performed using MrBayes v3.1.2 [39] under a GTR + I + Γ model as determined by jModelTest [40]. BI analyses consisted of two parallel runs of three incrementally heated chains and one cold chain each, and 3 million generations with sampling every 1000 generations. A burnin value of 750 was determined using TRACER V1.4 [41].

### 3.4. Metabolite Analysis, Data Processing and Multivariate Analysis

#### 3.4.1. GC-MS Analysis

GC-MS analyses of the non-polar eluates were performed using an Agilent 6890 gas chromatograph and Agilent 6973 mass selective detector. Sample injection volume was 1 μL. Split injection with a split ratio of 20:1 was used. The carrier gas was helium with a total flow rate of 72.7 mL·min^−1^ and 26.20 psi column head pressure. Compounds were separated using a 30 m × 0.25 mm HP-5 MS non-polar capillary column (Hewlett-Packard, 5% phenyl methyl siloxane, 0.25 μm thickness) for a run time of 67 min. under the following oven temperature program: 50 °C initial held for 2 min, then increased at a rate of 5 °C·min^−1^ to 300 °C, held for 15 min. The spectrometers were run in electron-impact mode with ionization energy of 70 eV and an ion source temperature ranging 230–250 °C. The scan range was set to detect masses 50–500 amu.

#### 3.4.2. GC-MS Data Processing

The software AMDIS [42] was used for peak identification and deconvolution of the chromatogram. This method calculates and retrieves pure (background-free) mass spectra from raw GC-MS data files based from the parameters indicated by the user. The following parameters were used for all of the analyses: medium shape requirement, medium sensitivity, and medium resolution. The ELU files generated from AMDIS were submitted for analysis using Spectconnect [43] to generate matrices of component peaks (relative abundance, retention time, integrated signal and base peak).

#### 3.4.3. Compound Identification

In the absence of pure standards of compounds previously isolated from *Portieria* (except for β-myrcene), we used KI’s, freely available mass spectral databases to determine identity of component peaks, and literature. We computed the Kovat’s or retention index of each peak using the retention times of alkane standards C8–C20 and the Retention Index Calculator [32]. The retention index is derived from the interpolation (usually logarithmic), relating the adjusted retention volume (time) of the sample component to the adjusted retention volumes (times) of two standards eluted before and after the peak of a sample component (IUPAC definition). The calculated KI was automatically compared to the KI’s of compounds stored in the built-in library of the calculator. Mass spectra of component peaks were compared to compounds retrieved from MassBank [44], Pherobase [45] and the limited version of the NIST library in the MS Search Program and AMDIS. Mass spectra of components were inspected for similarity with those of the characteristic mass signals of components published in [11,12,33].

#### 3.4.4. Data Standardization, Metabolite Fingerprinting and Multivariate Analyses

Relative abundance matrices generated from the Spectconnect analyses were used for statistical analyses. A matrix contains relative abundance values of all detected components across all samples included in the analysis. The data were standardized by obtaining a ratio between the relative abundance of a component per replicate (or sample) and the total of all components per replicate (or sample). The ratios obtained among the technical replicates were subsequently averaged. Principal component analysis was performed using Statistica 7.0 (StatSoft Inc., Tulsa, OK, USA) on each dataset to detect any pattern or groupings based from the variables which are the component compounds.

## 4. Conclusions

*Portieria* species in the Philippines are a rich source of secondary metabolites. This diversity in secondary metabolites amounts to at least 302 various compounds. The majority of which are exotic, remain undescribed and therefore are not available in natural product databases. Our study demonstrates that metabolic fingerprinting presents a practical approach to disclose intra- and interspecific patterns of secondary metabolites. Variation in secondary metabolites occurs between, as well as within, *Portieria* species. Preliminary results, based on a relatively small sample size, demonstrate that extensive intraspecific variation in secondary metabolites occurs between life-history stages. Female gametophytes (202 compounds) are chemically richer compared to that of the males (102) and tetrasporophytes (106). No spatio-temporal patterns were evident among the datasets. These results suggest that life-history driven variations and possibly other microscale factors may have an important influence on the variation of secondary metabolites within *Portieria* species. More exhaustive sampling is needed to confirm the life-stage specific metabolic fingerprints. In addition, to determine whether the intraspecific variation of the analyzed part of non-polar metabolome remains lower than the interspecific variation, it will be necessary to expand the taxon sampling across different cryptic species.

## Supplementary

**Table S1.** List of compounds isolated from *Portieria* (syn. *Desmia* and *Chondrococcus*) *hornemannii*, based on previous studies.

Reference	Cpd #	Name	Mol. Formula	Description	Specimen Source
[ 18]	1	Myrcene	C _10_H _16_	acyclic halogenated monoterpene	Amami Is., Japan
	2	7-chloro-myrcene	C _10_H _15_Cl	"	"
	3	7-bromo-myrcene	C _10_H _15_Br	"	"
	4	( *Z*)-10-bromo-myrcene	C _10_H _15_Br	"	"
	5	( *E*)-10-bromo-myrcene	C _10_H _15_Br	"	"
	6	( *Z*)-10-bromo-7-chloro-myrcene	C _10_H _14_BrCl	"	"
	7	( *E*)-10-bromo-7-chloro-myrcene	C _10_H _14_BrCl	"	"
	8	3-chloro-7, ( *Z*)-10-dibromo-myrcene	C _10_H _13_Br _2_Cl	"	"
	9	( *Z*)-10-chloro-3,7-dibromo-myrcene	C _10_H _13_Br _2_Cl	"	"
	10	3-bromo-7-chloro-myrcene	C _10_H _14_BrCl	"	"
	11	7-bromo-10-chloro-myrcene	C _10_H _14_BrCl	"	"
	12	-	C _10_H _15_Br	cyclic halogenated monoterpene	"
[ 19]	1	chondrocole A	C _10_H _14_BrClO	"	Hawaii
	2	chondrocole B	C _10_H _14_BrClO	"	Hawaii
[ 46]	3	-	C _10_H _15_Cl _3_Br _2_	acyclic halogenated monoterpene	Black Point, Oahu, Hawaii
	4	-	C _10_H _16_Cl _3_Br	"	"
	5	-	C _10_H _15_Cl _2_Br	"	"
	6	-	C _10_H _16_Cl _2_Br _2_	"	Halona Blowhole Is, Oahu, Hawaii
	10	-	C _10_H _16_Cl _2_Br _2_	acyclic halogenated monoterpene	"
	11	6-bromo-2-chloromyrcene	C _10_H _15_ClBr	"	Black Point, Oahu, Hawaii
	12	chondrocole c	C _10_H _14_Br _2_O	cyclic halogenated monoterpene	"
	13	-	C _10_H _15_Cl _3_Br _2_	acyclic halogenated monoterpene	Hawaii
	14	*Z*-3-bromomethylene-7-methyl-1,6-octadiene	C _10_H _16_ClBr _2_	"	Halona Blowhole Is., Oahu, Hawaii
	15	-	C _10_H _14_Br _2_Cl _2_	cyclic halogenated monoterpene	"
[ 47]	1	myrcene	C _10_H _16_	acyclic halogenated monoterpene	Kada Coast, Wakayama Pref., Japan
	2	7-chloro-myrcene	C _10_H _15_Cl	"	"
	3	7-bromo-myrcene	C _10_H _15_Br	"	"
	4	3,7-dichloro-myrcene	C _10_H _14_C _l2_	"	"
	5	( *Z*)-10-bromo-3-methoxy-α-myrcene	C _11_H _17_OBr	"	"
	6	( *E*)-10-bromo-3-methoxy-α-myrcene	C _11_H _17_OBr	"	"
	7	3-bromo-7-chloro-myrcene	C _10_H _14_BrCl	"	"
	8	( *Z*)-10-bromo-1-methoxy-myrcene	C _11_H _17_OBr	"	"
	9	( *E*)-10-bromo-1-methoxy-myrcene	C _11_H _17_OBr	"	"
[ 48]		(−)-3-bromomethyl-3-chloro-7-methyl-1,6-octadiene	C _10_H _16_BrCl	"	Trincomalee (Foul Point) Sri Lanka
[ 49]		2-(1-chloro-2-hydroxyethyl)-4,4-dimethylcyclohexa-2, 5-dienone: a precursor of 4,5-dimethylbenzo [b] furan	**-**	**-**	**-**
[ 20]	1	(2 *Z*,6 *E*)-1,8-dichloro-3-chloromethyl-7-methylocta-2,6-diene	C _10_H _15_Cl3	acyclic halogenated monoterpene	Rib Reef, Great Barrier Reef, Australia
	2	( *E*)-1,2-dibromo-3-chloromethylene-7-methyloct-6-ene	C _10_H _15_Br _2_Cl	"	"
	3	( *Z*)-1-chloro-3-chloromethyl-7-methylocta-2,6-diene	C _10_H _16_Cl2	"	"
	4	( *Z*)-1,6-dichloro-3-chloromethyl-7-methylocta-2,7-diene	C _10_H _15_Cl3	"	"
	5	(2 R*,3(8) *E*,4 S*,6 R*)-6-bromo-2-chloro-1,4-oxido-3,(8)-ochtodene	C _10_H _14_BrClO	epimeric bicyclic monoterpene	"
	6	(2 S*,3(8) *E*,4 S*,6 R*)-6-bromo-2-chloro-1,4-oxido-3,(8)-ochtodene	C _10_H _14_BrClO	"	"
[ 50]		( *Z*)-3-bromo-8-chloro-6-chloromethyl-2-methylocta-1,6-diene (9,6-hydroxymethyl-2-methylocta-2,8-dien-6-ol	-	-	-
[ 21]	1	(2 *Z*)-6-bromo-3-chloromethyl-1,7-dichloro-7-methylocta-2-ene	C _10_H _16_Cl _3_Br	acyclic halogenated monoterpene	Nelly Bay, Magnetic Island, Queensland, Australia
	2	(2 *Z*,6 *E*)-3-chloromethyl-1-chloroocta-2,6-dien-8-al	C _10_H _14_OCl _2_	"	"
	3	3-methoxymethyl-6-methoxyl-7-methylocta-1,7(10)-dien-3-ol	C _12_H _22_ O _3_	"	"
	4	(2 *Z*,6 *S*)-3-chloromethyl-1-methylocta-2,7(10)-dien-6-ol	C _11_H _19_ O _2_Cl	"	"
	5	(2 *Z*,6 *S*)-3-chloromethyl-6-methylocta-2,7(10)-dien-1-ol	C _11_H _19_ O _2_Cl	"	"
[ 22]	1	**halomon/**6( *R*)-bromo-3( *S*)-(bromomethyl)-7-methyl-2,3,7-trichloro-1-octene	C _10_H _15_Br _2_Cl _3_	acyclic halogenated monoterpene	Chanaryan, Batan Is., Batanes, Philippines
	2	-	C _10_H _13_Cl	cyclic halogenated monoterpene	Banilad, Bacong, Negros Oriental, Philippines
[ 23]	2	isohalomon	C _10_H _15_Br _2_Cl _3_	acyclic halogenated monoterpene; isomeric with halomon	Chanaryan, Batan Is., Batanes, Philippines
	3	-	C _10_H _14_BrCl _3_	acyclic halogenated monoterpene	"
	4	-	C _10_H _14_Br _2_Cl _2_	"	"
	5	-	C _10_H _14_Br _3_Cl	cyclic halogenated monoterpene	"
	6	-	C _10_H _14_BrCl _3_	"	"
	7	-	C _10_H _13_Cl _3_	"	"
	8	-	C _10_H _15_BrCl _2_	acyclic halogenated monoterpene	"
	9	-	C _10_H _16_Br _2_Cl _2_	"	"
	10	-	C _10_H _16_BrCl	"	Halona Blowhole Is, Oahu, Hawaii
	11	-	C _10_H _15_BrCl	"	"
	12	-	C _10_H _14_BrCl	cyclic halogenated monoterpene	"
[ 8]	1	**apakaochtodene A/**6( S*)-bromo-1,4( S*),8( R*)-trichloro-2(Z)-ochtodene	C _10_H _14_Cl _3_Br	cyclic halogenated monoterpene	Apaka Point Beach, Guam
	2	**apakaochtodene B**/6( *S*)-bromo-1,4( *S*), 8( R*)-trichloro-2( *E*)-ochtodene	C _10_H _14_Cl _3_Br	cyclic halogenated monoterpene	Gun Beach; Double Reef; Pago Bay; Guam
[ 11]	1	**myrcene/**7-methyl-3-methylene-1,6-octadiene	C _10_ H _16_O	acyclic non-halogenated monoterpene	microplantlet culture, Double Reef NW Guam
	2	**10 *E* -bromomyrcene/** *E*-3-bromomethylene-7-methyl-1,6-octadiene	C _10_H _15_Br	"	"
	3	**10 *Z* -bromomyrcene/** *Z*-3-bromomethylene-7-methyl-1,6-octadiene	C _10_H _15_Br	"	"
	4	**10 *E* -bromo-3-chloro-α-myrcene/** *E*-3-bromomethylene-6-chloro-1,7-octadiene	C _10_H _14_BrCl	"	"
	5	-	C _10_H _14_Br _2_	"	"
	6	**apakaochtodene B/**6( *S)-*bromo *-*1,4( *S*), 8( R*)-trichloro-2( *E*)-ochtodene	C _10_H _14_BrCl _3_	cyclic halogenated monoterpene	"
	7	-	C _10_ H _16_O	non-halogenated monoterpene	"
	8	bromomyrcene isomer	C _10_H _15_Br	halogenated monoterpene	"
	9	**7-chloromyrcene/**2-chloro-3-methylene-7-methyl-1,6-octadiene	C _10_H _15_Cl	"	"
	10		C _10_H _14_Cl	"	"
	11	chloromyrcene derivative	C _10_H _16_Cl	"	"
	12	bromomyrcene isomer	C _10_H _15_Br	"	"
	13	chloromyrcene derivative	C _10_H _14_Cl	"	"
	14	-	C _10_H _18_Cl _2_	"	"
	15	-	C _15_H _18_ O _4_	sesquiterpene	"
[ 13]	5	1,2-dibromoochtoda-3(8),5-dien-4-one	C _22_H _12_Br _2_O	cyclic halogenated monoterpene	Cape Zampa, Okinawa, Japan
	6	1-bromo-2-chloroochtoda-3(8),5-dien-4-one	C _10_H _12_BrClO	"	"
	7	1,2-dichloroochtoda-3(8),5-dien-4-one	C _10_H _12_Cl _2_O	"	Gushichan coast, Okinawa, Japan
	8	(1 *Z*)-1-bromoochtoda-1,3(8),5-trien-4-one	C _10_H _11_BrO	"	Cape Zampa, Okinawa, Japan
	9	(1 *Z*)-1-chloroochtoda-1,3(8),5-trien-4-one	C _10_H _11_ClO	"	"
[ 12]	1	**halomon/**6( *R*)-bromo-3( *S*)-(bromomethyl)-7-methyl-2,3,7-trichloro-1-octene	C _10_H _15_Br _2_Cl _3_	acyclic halogenated monoterpene	Tolagniaro, Fort Dauphin, Madagascar
	2	-	C _10_H _13_ClBr	"	"
	3	-	C _10_H _14_Cl _2_Br	"	"
	4		C _10_H _15_Br _2_Cl _3_	"	"

**Table S2.** Compound peaks detected from GC-MS analysis of samples used for characterization of the life history stages of *P. hornemannii*. Identifications based on comparison of either Kovat’s Indices (KI) of compounds retrieved from the Retention Index Calculator [37] or mass spectral comparison of compounds retrieved from NIST or MassBase. Asterisk (*) indicates compounds with parent ions showing halogenated mass spectral patterns.

#	Sample	Base Peak		KI	KI Based ID		Mass Spectra Based ID	
			RT (min)		Nearest KI	Lucero *et al.* [ 37]	NIST	MassBase
1	F6_2	93	6.1863	994	993/994	beta-myrcene/6-methyl-5-hepten-2-ol	b-myrcene	b-myrcene
2	T1_1	104	6.5874	1008	1007	α-phellandrene	-	-
3	T1_2	104	6.5909	1008	1007	α-phellandrene	-	-
4	M1_1	91	6.7685	1016	1017	α-terpinene	Benzene, tert-butyl-	4-methylacetophenone
5	F3_3	79	6.9501	1023	1024	r-cymene	-	-
6	M1_2	79	6.9532	1023	1024	r-cymene	-	-
7	M1_3	91	7.017	1026	1025	p-cymene	carbonic acid	1-phenylpropan-2-one
8	M2_2	91	7.021	1026	1025	p-cymene	-	protopine
9	F3_2	91	7.0387	1027	1025	p-cymene	-	*N*-Methyl- *N*propagylbenzylamine
10	T2_1	57	8.0381	1063	1062	y-terpinene	-	-
11	M2_2	132	8.8894	1089	1089	p-mentha-2,4(8)diene	-	-
12	T2_1	117	9.5127	1110	1099	linalool	-	-
13	F4_3	117	9.8874	1124	1123	chrysanthenone	-	-
14	M1_3	117	9.9855	1128	1127	α-campholenal	-	-
15	M2_2	91	10.2042	1136	1127	α-campholenal	-	-
16	F2_2	91	10.2132	1136	1127	α-campholenal	-	Dimethirimol
17	T2_1	119	10.4565	1145	1145	camphor	-	Benzimidazole
18	M1_1	68	10.8826	1160	1158	isobomeol	-	-
19	F7_3	68	10.8845	1160	1158	isobomeol	-	-
20	T2_1	68	10.8866	1160	1158	isobomeol	-	-
21	F3_3	68	10.8873	1160	1158	isobomeol	-	-
22	F4_1	117	11.3242	1174	1171	ethyl-benzoate	-	-
23	F4_1	50	11.5177	1180	1180	m-cymen-8-ol	-	-
24	T2_2	127	11.5317	1180	1180	m-cymen-8-ol	-	-
25	T2_3	134	11.7619	1188	1187	p-cymen-8-ol	-	-
26	M2_2	105	12.0654	1197	1196	methylchavicol	-	3-cyanopyridine
27	F3_2	57	12.1827	1200	1203	n-decanal	3-Hexanone	-
28	F7_3	113	12.3674	1207	1207	verbenone	-	-
29	M1_2	113	12.3831	1208	1207	verbenone	-	-
30	T1_3	91	12.3874	1208	1207	verbenone	-	-
31	M2_2	93	12.6294	1217	1217	trans-carveol	-	-
32	M1_2	69	12.7588	1222	1219	trans-carveol	-	-
33	M1_1	55	12.8397	1225	1229	nerol	-	-
34	F7_3	67	13.1203	1236	1236	thymol methyl ether	-	-
35	M1_1	79	13.1266	1236	1236	thymol methyl ether	-	-
36	F5_2	93	13.127	1236	1236	thymol methyl ether	-	Beta-pinene; y-eudesmol
37	M2_2	93	13.32	1243	1243	carvone	-	-
38	T1_1	93	13.327	1243	1243	carvone	2-chloropropionyl chloride	-
39	T2_1	93	13.3305	1243	1243	carvone	-	-
40 *(Br)	F6_2	69	13.6916	1256	1256	geraniol	-	-
41	M1_1	91	13.6953	1256	1256	geraniol	-	
42	F2_1	69	13.6967	1256	1256	geraniol	-	
43	F5_1	69	13.6975	1256	1256	geraniol	-	-
44	M2_1	91	13.9837	1266	1263	( *E*)-2-decenal	-	*N*-Methyl- *N*propagylbenzylamine
45	F7_3	119	14.131	1271	1271	geranial	-	-
46	F2_3	119	14.1354	1271	1271	geranial	-	-
47	F3_2	69	14.4252	1281	1282	a-terpinen-7-al	-	-
48	M2_3	69	14.4368	1281	1282	a-terpinen-7-al	-	-
49	F5_1	91	14.6745	1289	1289	p-cymen-7-ol	-	-
50	T2_1	91	14.68	1289	1289	p-cymen-7-ol	-	-
51	F6_3	149	14.9157	1297	1297	perilla alcohol	-	Benzoylcholine
52 * (Cl)	F6_3	131	15.0641	1302	1302	trans-ascaridole	-	2-chloro-1-phenyl-2-butene
53	F6_1	166	15.3344	1313	1313	2 *E*,4 *E*-decadienal	-	2-chloro-1-phenyl-2-butene
54	F6_3	81	15.5368	1320	1313	2 *E*,4 *E*-decadienal	-	-
55	M2_2	81	15.5381	1321	1313	2 *E*,4 *E*-decadienal	-	-
56	T1_2	81	15.5481	1321	1313	2 *E*,4 *E*-decadienal	-	-
57	M2_2	67	15.6269	1324	1313	2 *E*,4 *E*-decadienal	-	1-Adamantanamine
58	M1_2	67	15.6354	1324	1313	2 *E*,4 *E*-decadienal	-	-
59	F3_1	57	15.7447	1329	1339	d-elemene	-	-
60	F2_1	57	15.7649	1329	1339	d-elemene	-	-
61	M2_2	91	15.8827	1334	1339	d-elemene	-	-
62	M1_2	91	15.8885	1334	1339	d-elemene	-	-
63	M2_2	139	16.5157	1357	1357	eugenol	-	-
64	F6_1	115	16.5278	1358	1357	eugenol	-	-
65	M2_3	166	16.5392	1358	1357	eugenol	-	-
66	M2_2	91	17.02	1375	1373	a-ylangene	-	-
67	M2_3	91	17.0254	1376	1373	a-ylangene	-	-
68	M1_2	91	17.0311	1376	1373	a-ylangene	-	-
69	T2_1	127	17.403	1389	1389	isolongifolene	-	Imidazole-4-acetate
70	T2_1	57	17.7476	1400	1399	1,7-di-epi-a-cedrene	-	-
71	M2_2	79	17.7625	1401	1402	methyleugenol	-	-
72	M2_3	127	17.7782	1402	1402	methyleugenol	-	-
73	T2_2	67	18.1041	1415	1415	*cis*-a-bergarnotene	-	-
74	T1_3	79	18.18	1418	1419	β-caryophyllene	-	-
75	F6_1	79	18.1866	1419	1419	β-caryophyllene	-	-
76	T1_1	79	18.1928	1419	1419	β-caryophyllene	Pyridine	-
77	F5_1	79	18.1934	1419	1419	β-caryophyllene	-	camphene
78	M1_2	79	18.1939	1419	1419	β-caryophyllene	-	-
79 * (Br)	F7_2	212	18.3193	1424	1420	β-caroyophyllene	-	-
80	F6_2	210	18.4564	1429	1420	β-caroyophyllene	-	-
81	M2_3	131	18.4673	1430	1420	β-caroyophyllene	-	-
82	M1_1	67	18.5462	1433	1435	trans-a-bergamotene	-	-
83	M1_2	67	18.5503	1433	1435	trans-a-bergamotene	-	-
84	F2_2	133	18.6084	1435	1435	trans-a-bergamotene	-	-
85	T2_2	69	18.6925	1439	1438	trans-a-bergamotene	-	-
86	F3_3	69	18.6982	1439	1440	a-guaiene	-	-
87	M2_3	69	18.7018	1439	1440	a-guaiene	-	-
88	M1_1	91	18.7475	1441	1440	a-guaiene	-	-
89	F4_2	69	18.8243	1444	1440	a-guaiene	-	-
90	F7_2	69	18.8273	1444	1440	a-guaiene	-	-
91	T1_3	69	18.8306	1444	1440	a-guaiene	-	-
92	F2_2	210	19.1226	1456	1455	a-humulene	-	-
93	F3_3	133	19.3339	1464	1464	a-acoradiene	-	
94	T2_2	105	19.4843	1469	1469	drima-7,9(11)-diene	-	-
95	T2_1	105	19.4852	1469	1469	drima-7,9(11)-diene	-	-
96	F7_2	131	19.4915	1470	1469	drima-7,9(11)-diene	-	-
97	F5_1	91	19.6255	1475	1474	b-cadinene	-	-
98	F6_3	91	19.7468	1479	1479	y-curcurnene	-	
99	F2_1	91	19.7581	1480	1480	germacrene D	-	-
100	F4_1	153	20.0567	1491	1491	*cis*-β-guaiene	-	-
101	F4_1	153	20.0567	1491	1491	*cis*-β-guaiene	-	-
102	F3_2	71	20.2111	1496	1495	a-zingiberene	-	
103	F7_2	135	20.3621	1502	1499	a-muurolene	-	-
104	F5_1	91	20.3727	1503	1506	d-selinene	-	-
105	T2_1	91	20.3766	1503	1506	d-selinene	-	-
106	F2_2	91	20.3829	1503	1506	d-selinene	--	-
107	F6_1	205	20.6221	1513	1513	g-cadinene	-	-
108	F4_3	205	20.6237	1513	1513	g-cadinene	-	-
109	F7_2	205	20.6252	1513	1513	g-cadinene	-	5-bromo-4-methoxyoct-1-ene
110	T2_1	205	20.6278	1513	1513	g-cadinene	p-Benzoquinone	-
111	F3_2	133	20.6781	1516	1514	sesquicineole	-	-
112	F3_3	133	20.6803	1516	1514	sesquicineole	-	-
113	F6_3	133	20.685	1516	1514	sesquicineole	-	-
114	F5_1	69	20.8293	1522	1523	d-cadinene	-	--
115	F5_1	69	20.9154	1526	1525	eugenyl acetate	-	-
116	F6_3	69	20.9158	1526	1525	eugenyl acetate	-	-
117	F7_2	121	20.9204	1526	1525	eugenyl acetate	-	-
118	T1_3	69	20.9227	1526	1525	eugenyl acetate	-	-
119	F7_2	167	21.1103	1534	1533	cadina-1,4-diene1	-	-
120	T2_2	91	21.1498	1536	1538	a-cadinene	-	-
121	F6_1	167	21.43	1547	1548	elemol	-	-
122	F7_2	167	21.4346	1547	1548	elemol	-	-
123	T1_3	69	21.467	1549	1549	elemol	-	--
124	F3_2	69	21.4709	1549	1549	elemol	-	-
125	F7_2	91	21.598	1554	1552	elemicin	-	-
126	F6_2	91	21.6852	1557	1557	germacrene B	-	-
127	F5_1	91	21.6859	1558	1557	germacrene B	-	-
128	F6_3	91	21.6869	1558	1557	germacrene B	-	-
129	M1_1	91	21.6895	1558	1557	germacrene B	-	-
130	F6_3	67	21.7268	1559	1557	germacrene B	-	-
131	F7_2	133	21.8253	1563	1564	β-calacorene	-	l-Asparagine
132	T1_1	91	21.8405	1564	1564	β-calacorene	-	-
133	F7_2	167	22.0012	1570	1574	prenopsan-8-ol	-	-
134	T1_3	213	22.018	1571	1574	prenopsan-8-ol	-	-
135	F6_3	132	22.0226	1571	1574	prenopsan-8-ol	-	-
136	F6_3	153	22.0226	1571	1574	prenopsan-8-ol	-	-
137	M1_1	91	22.3956	1586	1585	gleenol	-	-
138	F2_2	133	22.5362	1591	1590	viridflorol	-	-
139	F6_3	71	22.7179	1598	1590	viridflorol	-	-
140	F3_2	71	22.7193	1598	1590	viridflorol	-	-
141	F2_3	135	22.722	1598	1590	viridflorol	-	-
142	F3_3	57	22.7547	1600	1607	b-oplopenone	-	-
143	F3_2	57	22.7603	1600	1607	b-oplopenone	-	-
144	F3_3	57	22.761	1600	1607	b-oplopenone	-	-
145	F6_3	67	22.8782	1605	1607	b-oplopenone	-	-
146	T2_2	68	23.0541	1613	1612	tetradecanal	-	-
147	F4_3	148	23.2435	1622	1623	silphiperfol-6-en-5-one	-	-
148	F3_1	135	23.2485	1622	1623	silphiperfol-6-en-5-one	-	-
149	F5_3	91	23.52	1634	1633	y-eudesmol	-	-
150	F4_3	91	23.5226	1634	1633	y-eudesmol	-	-
151	F7_2	68	23.5261	1634	1633	y-eudesmol	-	-
152	F7_2	68	23.5261	1634	1633	y-eudesmol	-	--
153	T2_2	68	23.5278	1634	1633	y-eudesmol	-	-
154	F6_1	167	23.5309	1634	1633	y-eudesmol	-	
155	F6_2	67	23.7239	1643	1642	cubenol	-	allylcy-anide
156	T1_3	91	23.8325	1647	1646	a-muurolol	-	-
157	F7_2	132	23.8759	1649	1646	a-muurolol	-	-
158	F7_2	132	23.9988	1654	1653	a-cadinol	-	-
159	F6_2	149	24.3377	1669	1668	bulnesol	-	-
160	F7_2	149	24.3428	1669	1668	bulnesol	-	-
161	F6_3	167	24.6091	1680	1682	a-bisabolol	-	-
162	F6_1	57	24.6252	1681	1682	a-bisabolol	-	-
163	F7_2	69	24.8113	1689	1686	8-cedren-13-ol	-	-
164	F7_2	69	24.8207	1689	1686	8-cedren-13-ol	-	-
165	F2_3	67	24.9282	1693	1686	8-cedren-13-ol	-	-
166	F6_1	57	25.0854	1700	1686	8-cedren-13-ol	-	Heptadecane
167	F7_2	91	25.3006	1710	1686	8-cedren-13-ol	-	-
168	F3_2	71	25.3224	1711	1735	oplopanone	-	-
169	F3_3	68	25.4814	1718	1735	oplopanone	-	-
170	F6_3	67	25.7278	1730	1735	oplopanone	-	-
171	F7_2	103	25.7292	1730	1735	oplopanone	-	-
172	F2_1	67	25.7318	1730	1735	oplopanone	-	-
173	F2_3	133	25.8305	1735	1735	oplopanone	-	-
174	F3_1	117	25.8366	1735	1735	oplopanone	-	-
175	F7_2	167	26.3223	1757	1761	benzyl-benzoate	-	-
176	T2_1	167	26.5838	1769	1761	benzyl-benzoate	-	6-Methylmer-captopurine
177	F6_2	117	26.7333	1775	1789	8-a-acetoxyelemol	-	-
178	T2_2	103	26.8111	1779	1789	8-a-acetoxyelemol	-	-
179	F3_1	67	26.8577	1781	1789	8-a-acetoxyelemol	-	-
180	F6_1	67	26.8599	1781	1789	8-a-acetoxyelemol	-	-
181	F3_2	67	26.8671	1781	1789	8-a-acetoxyelemol	-	-
182	T2_1	67	26.8697	1781	1789	8-a-acetoxyelemol	-	-
183 * (Cl _2_)	F6_1	103	26.8838	1782	1789	8-a-acetoxyelemol	-	-
184	F3_1	103	26.8852	1782	1789	8-a-acetoxyelemol	-	-
185 * (Br)	F6_1	67	27.4286	1806	1798	nootkatone	-	-
186 * (Br)	F7_2	67	27.4342	1806	1798	nootkatone	-	-
187	F2_1	67	27.4383	1807	1798	nootkatone	-	-
188	F6_3	103	27.4961	1810	1798	nootkatone	-	-
189	F6_1	67	27.5012	1810	1798	nootkatone	-	-
190	F6_2	67	27.5676	1813	1827	isopropyl tetradecanoate	-	-
191	F6_2	69	28.0351	1836	1827	isopropyl tetradecanoate	-	-
192	F3_2	69	28.0387	1836	1827	isopropyl tetradecanoate	-	-
193	F6_1	91	28.3153	1849	1867	flourensiadiol	-	-
194	F3_2	149	28.735	1869	1867	flourensiadiol	-	-
195 * (ClBr)	F6_2	69	29.2918	1895	1878	hexadecanol	-	-
196	F5_3	67	29.4766	1903	1927	methyl hexadecanoate	-	-
197	T2_1	67	29.4889	1904	1927	methyl hexadecanoate	-	-
198	F7_2	91	30.7705	1968	1999	eicosane	-	-
199	T2_2	91	30.7753	1969	1999	eicosane	-	-
200	F3_2	91	30.7855	1969	1999	eicosane	-	-
201	T2_2	64	31.6475	-	-	-	-	-
202	T2_2	129	38.661	-	-	-	-	-

**Table S3.** Compound peaks detected from GC-MS analysis of samples for evaluation of non-polar secondary metabolite patterns of 5 cryptic species of *P. hornemanni* found in Batanes, Philippines. Identifications based on comparison of either Kovat’s Indices (KI) of compounds retrieved from the Retention Index Calculator [37] or mass spectral comparison of compounds retrieved from NIST or MassBase. Asterisk (*) indicates compounds with parent ions showing halogenated mass spectral patterns.

#	Sample	Base Peak		KI	KI Based ID		Mass Spectra Based ID	
			RT (min)		Nearest KI	Lucero *et al.* [ 37]	NIST	Massbase
1	B4B	57	8.1122	1065	1062	y-terpinene	-	-
2	B4C	69	8.7386	1085	1085	artemisia-alcohol	-	-
3	B4C	57	9.1807	1098	1097	linalool	-	-
4	B4B	71	9.1841	1098	1097	linalool	-	-
5	B6C	57	9.4047	1106	1099	linalool	3-hexanone, 2,2-dimethyl-	2-methylbutane
6	B4C	69	11.2515	1172	1171	ethyl-benzoate	-	-
7- * (Cl)	B3A	69	11.2560	1172	1171	ethyl-benzoate	-	-
8	B4A	57	14.3547	1279	1277	trans-carvone-oxide	-	2,2-dimethylbutane
9	B4B	57	14.3593	1279	1277	trans-carvone-oxide	-	3-ethylhexane
10	B3B	57	14.5560	1285	1286	borneol-acetate	-	
11	B3A	69	15.2678	1310	1306	undecanal	-	trans-4-octene
12	B3A	57	15.6497	1325	1314	2 *E*,4 *E*-decadienal	-	
13	B6B	57	15.6518	1325	1314	2 *E*,4 *E*-decadienal	-	
14	B4A	71	16.5884	1360	1357	eugenol	-	2-methylpentane
15	B6C	57	17.8154	1403	1403	italicene	-	2,2-dimethylbutane
16	B3A	57	17.8289	1404	1403	italicene	-	
17	B10	71	20.306	1500	1499	a-muurolene	-	Pentacosane
18	B3A	57	20.4025	1504	1506	d-selinene	-	tripropylamine
19	B4A	191	20.715	1517	1514	sesquicineole	-	
20	B6C	57	21.0008	1529	1532	cadina-1,4-diene	-	
21- * (Br)	B3B	57	21.0024	1529	1532	cadina-1,4-diene	-	1-octene
22	B4A	69	21.5566	1552	1552	elemicin	-	
23	B4A	57	25.1819	1704	1686	8-cedren-13-ol	-	
24	B4B	71	25.4142	1715	1735	oplopanone	-	Hexacosane
25	B4B	69	26.0498	1745	1747	6 *S*-7 *R*-bisabolone	-	10-methyl-1-dodecanol
26	B4A	57	27.4031	1805	1798	nootkatone	-	4-methyl-1-pentene
27	B3B	57	27.9515	1832	1827	isopropyl-tetradecanoate	-	2,4-dimethylpentane
28	B3B	71	29.9932	1930	1927	methyl-hexadecanoate	-	docosane
29	B6B	57	30.4182	1951	1927	methyl-hexadecanoate	-	-
30	B10	69	30.5987	1960	1927	methyl-hexadecanoate	-	-
31	B4C	73	30.6949	1965	1999	eicosane	-	-
32	B6B	71	32.0267	-		-	3-hexanone, 2,2-dimethyl-	2,2-dimethylbutane
33	B3B	71	32.0458	-		-	-	2,2-dimethylbutane
34	B6B	69	34.1452	-		-	-	docosane
35	B3B	69	34.7128	-		-	-	-
36	B4C	69	34.7233	-		-	-	-
37	B6C	71	35.0713	-		-	-	-
38	B3C	71	36.7162	-		-	-	3-ethylpentane
39	B9B	395	38.2697	-		-	-	-
40	B6C	71	38.8875	-		-	-	3-ethylhexane
41	B6B	71	39.0147	-		-	-	-
42	B4C	57	39.4792	-		-	-	3-ethylhexane
43	B6B	57	39.4822	-		-	-	3,3-dimethyl-1-butene
44	B4C	71	39.6266	-		-	-	3-ethylpentane
45	B3C	71	39.7884	-		-	-	-
46	B6B	57	40.4997	-		-	3-hexanone, 2,2-dimethyl-	2-methylbutane
47	B6C	57	40.7684	-		-	-	3-ethylpentane
48	B3C	71	40.7722	-		-	-	-
49	B4C	57	43.09	-		-	-	3-ethylpentane
50	B6B	57	43.0982	-		-	-	2,2,4,6,6-pentamethylheptane
51	B6C	85	43.2865	-		-	-	2-methylnonane
52	B6B	71	43.3628	-		-	-	3-ethylpentane
53	B3C	71	43.3691	-		-	-	-
54	B3B	85	43.7727	-		-	-	3,3-dimethyl-1-butene
55	B4B	85	44.3901	-		-	-	Amitrole
56	B6B	71	44.3953	-		-	-	3-aminopropiononitrile
57	B4A	71	46.2638	-		-	-	-
58	B3C	71	46.8143	-		-	-	2,3-dimethylbutane
59	B6B	71	46.822	-		-	3-hexanone, 2,2-dimethyl-	-
60	B6C	71	47.3544	-		-	-	-
61	B4A	71	47.7467	-		-	-	3-ethylypentane
62	B3A	57	47.7529	-		-	-	3,3-dimethyl-1-butene
63	B4A	71	48.0072	-		-	-	-
64	B6B	71	48.0159	-		-	-	-
65	B6C	71	48.0169	-		-	-	-
66	B4A	71	48.0321	-		-	-	-
67	B4B	71	50.7731	-		-	-	-

**Table S4.** Compound peaks detected from GC-MS analysis of samples used for evaluation of temporal and spatial patterns of non-polar metabolites of three cryptic species of *P. hornemanni* found in the Visayas. Identifications based on comparison of either Kovat’s Indices (KI) of compounds retrieved from the Retention Index Calculator [37] or mass spectral comparison of compounds retrieved from NIST or MassBase. Asterisk (*) indicates compounds with parent ions showing halogenated mass spectral patterns.

#	Sample	Base Peak		KI	KI Based ID		Mass Spectra Based ID
			RT		Nearest KI	Lucero *et al.* [ 37]	Massbase
1	3A	57	8.1118	1065	1062	y-terpinene	undecane
2	15A	57	14.556	1285	1286	borneol acetate	1-nonene
3	9A	57	14.556	1285	1286	borneol acetate	pentacosane
4	29A	57	20.3128	1500	1499	a-muurolene	tricosane
5	15B	71	20.5458	1510	1509	β-bisabolene	3-ethylpentane
6	15A	57	25.186	1705	1686	8-cedren-13-ol	-
7	34A	57	25.4206	1716	1735	oplopanone	-
8	15B	71	26.6674	1772	1761	benzyl-benzoate	2-methylbutane
9	15A	57	27.8534	1827	1827	isopropyl tetradecanoate	
10	44BB	71	30.0015	1930	1927	methyl hexadecanoate	docosane
11	8B	57	30.4069	1950	1927	methyl hexadecanoate	
12	15B	71	31.6111	-	-	-	-
13	15A	57	31.8326	-	-	-	-
14	15B	57	31.8342	-	-	-	1,4-dimethylhexane
15	44BA	71	34.1535	-	-	-	docosane
16	15B	71	34.2967	-	-	-	3-ethylpentane
17	15A	57	34.76	-	-	-	1-octene
18	15A	71	35.4496	-	-	-	2-methylbutane
19	15B	57	35.5757	-	-	-	-
20	15B	57	35.5757	-	-	-	2,2-dimethylbutane
21	44BA	71	37.9407	-	-	-	docosane
22	15B	410	38.2743	-	-	-	-
23	15A	71	39.3484	-	-	-	3-ethylhexane
24	15A	71	40.0877	-	-	-	2,2-dimethylbutane
25	15A	71	40.0981	-	-	-	beta-Aminopropionitrile
26	15B	71	42.5358	-	-	-	2-methylbutane
27	15B	57	43.9626	-	-	-	4-methyloctane
28	44C	71	44.6454	-	-	-	-
29	15A	57	45.2255	-	-	-	2,2-dimethylbutane
30	15A	71	47.3529	-	-	-	-
31	15B	57	47.3534	-	-	-	-
32	15B	57	47.3581	-	-	-	2,4-dimethylpentane
33	15A	71	47.6237	-	-	-	2,4-dimethylhexane

Figure S1. Map of sampling sites. (**a**) Map of the Philippines indicating location of Batanes and Visayas; (**b**) Sampling sites in Batan and Sabtang Islands in Batanes; (**c**) Sampling sites in Siquijor, Negros, and Cebu Islands in the Visayas).

## Figures and Tables

**Table 1:** Frequency of compounds that are unique to or common to several species of *Portieria* found in Batanes. Values in parenthesis indicate total number of compounds found in a species, while 1 indicates presence and 0 absence of a compound.

B21 (49)	B33 (51)	B35 (60)	B8 (57)	B5 (57)	Frequency (67)
0	0	0	1	0	1
0	0	1	1	0	3
0	0	1	1	1	3
0	1	0	0	0	1
0	1	0	1	0	2
0	1	1	1	0	1
0	1	1	1	1	7
1	0	1	0	1	3
1	0	1	1	0	2
1	0	1	1	1	4
1	1	0	0	1	2
1	1	0	1	1	1
1	1	1	0	1	4
1	1	1	1	1	33
