# Supplementary figures and images for: Variability of Non-Polar Secondary Metabolites in the Red Alga Portieria
Source: Mar Drugs. 2011 Nov 21;9(11):2438–68. doi: 10.3390/md9112438 (PMC3229244; doi:10.3390/md9112438)

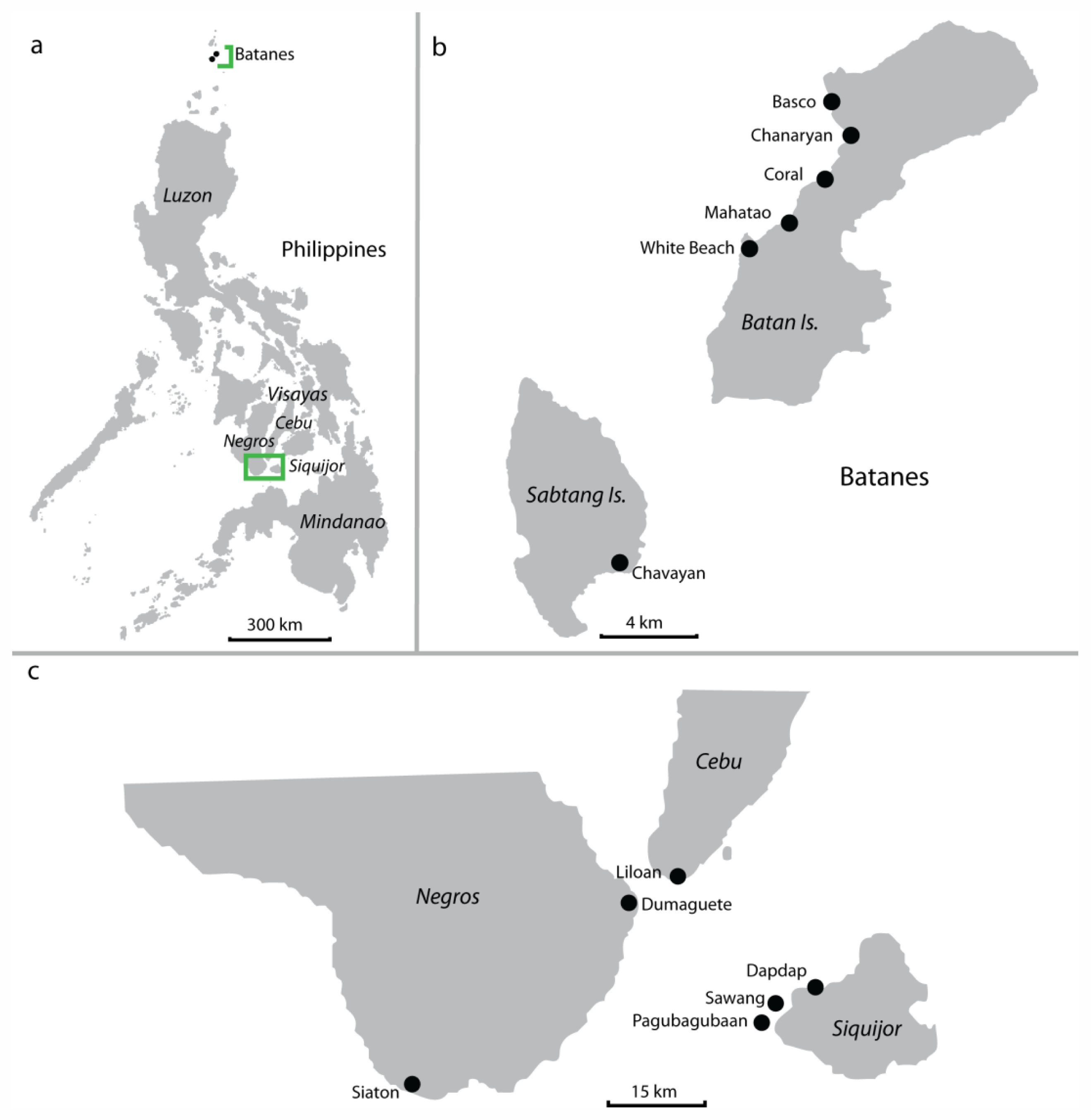

Supplement: Figure S1. — Map of sampling sites. (a) Map of the Philippines indicating location of Batanes and Visayas; (b) Sampling sites in Batan and Sabtang Islands in Batanes; (c) Sampling sites in Siquijor, Negros, and Cebu Islands in the Visayas). [file marinedrugs-09-02438s1.tif]
